# A28 TIME-RESTRICTED FEEDING REDUCES INFLAMMATORY BOWEL DISEASE AND IMPROVES EPITHELIUM COLON REGENERATION

**DOI:** 10.1093/jcag/gwac036.028

**Published:** 2023-03-07

**Authors:** V Carmona Alcocer, Z Taleb, A Todorovski, J Macdonald, T Igbokwe, P Karpowicz

**Affiliations:** 1 Department of Biomedical Sciences, University of Windsor, Windsor, Canada

## Abstract

**Background:**

The circadian clock is a time keeping mechanism that drives daily rhythms in the body, including behavior and gastrointestinal digestion, inflammation, and metabolism. These daily rhythms are endogenous to cells, and they must adjust themselves to cyclic environmental cues like feeding schedules and light dark cycles. However, when the cyclical environment is chrono-disrupted (ex. shiftwork and jetlag), the temporal organization in the body is lost. Chrono-disruption is linked to an increase in the risk of Inflammatory Bowel Disease (IBD); studies in rodents shown that IBD is more severe in mice without a circadian clock and intestinal regeneration is compromised

**Purpose:**

Recent studies have shown that time-restricted feeding (TRF, the provision of food at times of peak behavior activity – without caloric restriction), that aligns with circadian behavior improves daily organization in the body and health. Therefore, we hypothesized that time-restricted feeding would be an effective strategy to ameliorate IBD and improve regeneration in chrono-disrupted animals.

**Method:**

To test this, we used the DSS induction of colitis in mutant mice without clock function (*Bmal1*^*-/*-^) and controls with an intact clock (*Bmal1*^*+/+*^) and compared *ad libitum* feeding *vs*. TRF conditions (food available from 7pm to 7am).

**Result(s):**

Our results show that TRF ameliorates IBD symptoms and protects against epithelium colon damage in animals without clock function. Our preliminary results suggest that TRF may function by increasing the expression of the anti-inflammatory signal PPARg in the colon. Currently, we are testing if the circadian clock controls the daily expression of *PPARg in vivo* and *in vitro*. To further test the effect of TRF during regeneration after colitis, we tested proliferation (Ki67) and regeneration in the colon. (*Hopx*). Our results show that TRF increases the number of Ki67 positive cells and the expression of fetal-like regenerative precursor *Hopx* in both animals with and without a functional clock.

**Conclusion(s):**

Together these data suggest that TRF enhances regeneration in the colon after colitis. Moving forward, we are testing transcriptional pathways that are activated during regeneration in animals under TRF. Our results show that time restricted feeding can ameliorate IBD and highlight the role of circadian rhythms in regeneration and intestinal stem cell biology.

**Please acknowledge all funding agencies by checking the applicable boxes below:**

CIHR, Other

**Please indicate your source of funding;:**

Crohn's and Colitis Canada

**Disclosure of Interest:**

None Declared

